# Theaflavin promoted apoptosis in nasopharyngeal carcinoma unexpectedly via inducing autophagy *in vitro*

**DOI:** 10.22038/IJBMS.2021.59190.13143

**Published:** 2022-01

**Authors:** Jing Xu, Shu-Juan Wang, Shan-Shan Bu, Xiao-Qi Guo, Hong Ge

**Affiliations:** 1 Department of Radiotherapy, The Affiliated Cancer Hospital of Zhengzhou University, Zhengzhou, Henan province, China

**Keywords:** Apoptosis, Autophagy, Nasopharyngeal carcinoma, Proliferation, Theaflavin

## Abstract

**Objective(s):**

This study aimed to investigate the mechanism of the anticancer effect of theaflavin (TF) in nasopharyngeal carcinoma.

**Materials and Methods:**

CNE2 cells were used to study the anticancer effect of TF. This study used Cell Counting Kit-8 (CCK8) assay on proliferation and used flow cytometry to detect apoptosis. The protein expression of Bcl-2, Bax, caspase 3, and caspase 9 was detected by Western blot, and autophagy-related proteins were also detected.

**Results:**

TF inhibited proliferation of CNE2 cells, promoted apoptosis, and up-regulated the expression of caspase 3, caspase 9, and Bax, and decreased the level of Bcl-2. Unexpectedly, TF induced autophagy rather than inhibiting autophagy through up-regulating the levels of the autophagy marker light chain 3 (LC3) and Lysosomal-associated membrane protein 1 (LAMP1) and reducing levels of the autophagosome cargo protein p62, and the effect was via the mTOR pathway. Besides, autophagy inhibitor Chloroquine (CQ) suppressed the effect of TF on Bax, Bcl-2 and activation of caspase 3 and caspase 9.

**Conclusion:**

TF promoted apoptosis of nasopharyngeal carcinoma cells, the mechanism was unexpectedly involved in inducing autophagy.

## Introduction

Nasopharyngeal carcinoma is an uncommon cancer generated from the nasopharynx epithelium. This cancer has a different kind of differentiation and can usually be found at Rosenmüller’s fossa. According to the WHO categorization, it can be classified into three groups: 1. Non-keratinizing differentiated type; 2. Non-keratinizing undifferentiated type; and 3. Keratinizing type (1). In the development of nasopharyngeal carcinoma, in addition to genetic predisposition and Epstein-Barr virus infection, environmental factors and diet are also considered to play important roles (2, 3). Therefore, it is of great significance to study the pathogenesis and prevention of nasopharyngeal carcinoma.

Radiotherapy and chemotherapy are effective methods for nasopharyngeal carcinoma. However, patients with advanced and well-differentiated nasopharyngeal carcinoma have low sensitivity to radiotherapy and poor prognosis(4). Therefore, there is an urgent need to seek more efficient treatment methods and drugs for the clinical treatment of nasopharyngeal carcinoma.

Polyphenols are found in many plants and have many anticancer properties, including inhibition of cancer cell proliferation, tumor growth, angiogenesis, metastasis, inflammation, and induction of cell apoptosis. Theaflavin is a polyphenol compound that has many biological activities, such as antitumor, antivirus, and anti-free radical oxidation activities, and inhibits lipid peroxidation. In recent years, it has been found that theaflavin (TF) can inhibit the proliferation of a series of human tumor cells by inducing apoptosis (5-10).

Autophagy is a type of programmed cell death that parallels apoptosis and necrosis (11). Recent studies have found that abnormal autophagy signaling pathways are closely related to the occurrence and development of tumors (12, 13). Generally, autophagy impedes the induction of apoptosis, however, it may play a useful role in inducing apoptosis in some special cases (14). Moderate autophagy allows cells to adapt to unfavourable factors, which is beneficial to the survival of tumor cells, but excessive autophagy can damage organelles and cause autophagic cell death (15, 16). Therefore, autophagy has also become a research hotspot as a common target for new treatments for tumors and other diseases (17-20). 

Recent studies have found that theaflavins can induce autophagy (21). The effect of TF on autophagy in nasopharyngeal carcinoma cells has not been reported. Therefore, it is important to elucidate the antitumor effects of TF on nasopharyngeal carcinoma and its possible mechanism. In this study, we studied the effects of theaflavins on the proliferation and autophagy of nasopharyngeal carcinoma CNE2 cells. The possible mechanism of its anti-nasopharyngeal carcinoma was further explored to provide a theoretical basis for the research and application of theaflavins in the treatment of nasopharyngeal carcinoma. 

## Materials and Methods

***Chemicals and reagents***

Theaflavin (No. A0954, CAS: 4670-05-7 3, purity ≥ 98%, Chengdu Must Biotechnology Co., Ltd. Chengdu, China) and Cell Counting Kit 8 (WST-8/CCK-8) (ab228554) were purchased from Abcam. Dulbecco’s modified Eagle’s medium (DMEM) and chloroquine (C6628) were purchased from Sigma-Aldrich. Torin1 (2273–5) was purchased from Bio Vision Inc. Fetal bovine serum (10270–106) was purchased from Life Technologies. SDS (L3771) was purchased from Sigma-Aldrich. The Clonogenic Assay Kit (CA-001, Biopioneer), Apoptosis Kit (V13242, Thermo Fisher), Lipofectamine™ 3000 Transfection Reagent (L3000075, Thermo Fisher), and LC3, p62, LAMP1, mTOR, p-mTOR, P70S6K, p-P70S6K, caspase 3, caspase 9 and β-actin antibodies were all purchased from Cell Signaling Technology.

***Cell viability assay***

To evaluate cytotoxicity, a Cell Counting Kit-8 assay was performed to evaluate cell viability. CNE2 cells were seeded into 96-well culture plates and incubated with theaflavin (5, 10, 20, 40, 80, or 160 μM) for 24, 48, or 72 hr. Then, 10 μl of CCK-8 was added, and the cells were incubated for another 3 hr. The absorbance of CCK-8 was measured at 450 nm.

***Colony formation assay***

CNE2 cells were cultured to the logarithmic growth phase, put into a 6-well plate to attain a cell density of 500 cells per well, and placed into a CO_2_ incubator for culture. After 24 hr, different concentrations of TF (20, 40, or 80 μM) were added, and the culture medium was changed every 2 days. After 2 weeks, the medium was discarded, washed twice with PBS, fixed with 4% paraformaldehyde for 15 min, and stained with crystal violet for 30 min. The colony formation number was calculated.

***Cell apoptosis assay***

CNE2 cells were treated with different concentrations of theaflavin (20, 40, or 80 μM) for 48 hr. The cells were collected and washed with phosphate-buffered saline (PBS) 3 times. Then, the cells were stained with a propidium iodide annexin V FITC apoptosis detection kit. Finally, apoptosis was analyzed by flow cytometry (BD Biosciences, Franklin Lakes, NJ).

Terminal deoxynucleotidyl transferase dUTP nick end labeling (TUNEL) assay

CNE2 cells were treated with Sepin-1 for 24 hr and then separated and rotated into slides. The cells were then fixed, permeabilized, and labeled by following the protocol for the ApopTag ISOL Dual Fluorescence Apoptosis Detection Kit (DNase Types I & II). The slides were examined under a fluorescence microscope.

***Immunoblotting***

The cells were first rinsed on ice with cold PBS and then lysed in ice-cold 1x lysis buffer with a phosphatase inhibitor and protease inhibitor mixture. The soluble contents extracted from cell lysates were isolated by centrifugation at 14000 rpm for 30 min in a centrifuge. To determine the p62 levels, 1% SDS was mixed into lysis buffer to identify the whole cellular pool. The proteins were denatured by boiling at 95 **°**C for 10 min, isolated by 12% to 18% SDS-PAGE, and transferred to a nitrocellulose membrane. The membrane was then blocked in TBS-T buffer with 5% nonfat milk (TBS containing 0.05% Tween-20) and incubated overnight at 4 **°C **with primary antibodies** (**LC3, 1:5000; p62, 1:3000; LAMP1, 1:1000; mTOR, 1:1000; p-mTOR, 1:1000; P70S6K, 1:1000; p-P70S6K, 1:500; caspase 3, 1:1000; caspase 9, 1:1000 and β-actin, 1:10000). Then, the membrane was incubated for 1 hr using secondary antibodies. The ECL kit was used to test the protein signals and analyze the results.

***RFP-GFP-LC3 plasmid transfection and immunofluorescence assays***

Cells in 24-well plates were plated on coverslips. CNE2 cells were transfected with the RFP-GFP-LC3 plasmid using Lipofectamine 3000 following the protocol. The cells were treated with TF for 48 hr after transfection for 24 hr. The coverslips were rinsed three times with PBS after treatment with TF, fixed with 4% paraformaldehyde (PFA) for 10 min, permeated with 0.25% Triton X-100 for 10 min, and blocked with 1% BSA in PBS for 1 hr. After three washes, the slices were then stained with DAPI. Under an Eclipse 80i fluorescence microscope, the number of LC3 puncta per cell was calculated and quantified.

***Statistical analysis***

Statistical analysis was performed using GraphPad Prism 8.0 software, each value corresponds with data from three independent experiments, expressed as the mean ± standard deviation (SD). One-way ANOVA was used for comparisons between groups, and Tukey’s test was used for multiple comparisons. Significance was set at *P*<0.05.

## Results

***Effects of TF on the viability of CNE2 cells***

**Figure 1:**
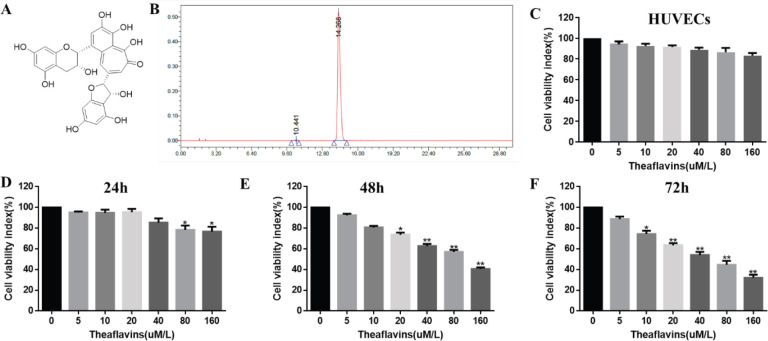
Effect of theaflavin (TF) on the viability of CNE2 cells. (A) Structure of TF. (B) Chromatogram of TF detected by high-performance liquid chromatography (HPLC). (C, D, E, F) Cell viability was tested by the CCK-8 assay method. (C) Effect of TF on normal cells (HUVECs) for 72 hr, (D-F) Effect of TF on CNE2 cells. The data are presented as the mean ± SD from three independent experiments. *P<0.05, **P<0.01, compared with the control group

As shown in Figure 1, there was less toxicity of TF on normal cells (Human umbilical vein endothelial cells, HUVECs); and the viability of CNE2 cells was detected by a CCK-8 kit. Cells were treated with different dosage of TFs (0, 5, 10, 20, 40, 80, or 160 μM) at different times (24, 48, or 72 hr). Our results revealed that the CNE2 cells were remarkably inhibited in a dose dependent manner and this inhibitory effect reached a peak at the dose of 160 μM (76.67% of the ctrl group). Besides, 20 and 40 μM TF significantly inhibited CNE2 cell viability at 48 and 72 hr and 80, and 160 μM TF markedly suppressed the proliferation of CNE2 cells at all time 

***Effects of TF on the cell colony formation ability of CNE2 cells***

**Figure 2:**
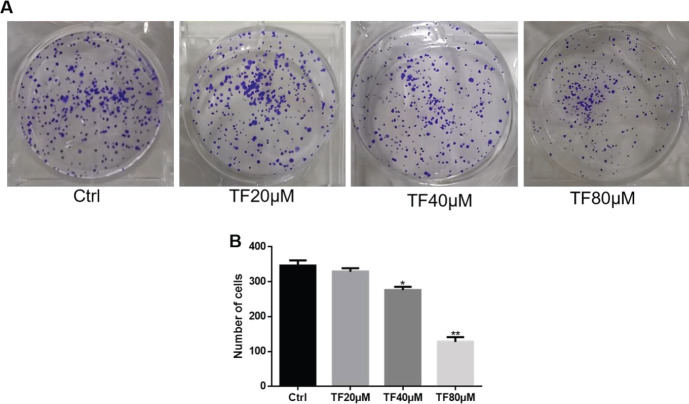
Effect of theaflavin (TF) on the cloning ability of CNE2 cells detected by a cell colony formation test. (A) Colonies were formed by CNE2 cells after treatment with TF at different concentrations (20, 40, or 80 μM). (B) Data are presented as the mean ± SD from three independent experiments. *P<0.05 and **P<0.01, compared with the control group

As shown in Figure 2, the effect of TF on the colony formation ability of CNE2 cells was detected. Consistently, the results revealed that the number of colonies formed was reduced when the cells were treated with TF in a dose-dependent manner, and the high concentrations (40 and 80 μM) generated a significant result compared with the control group, the number of clones reduced by 20.39% and 63.17%, respectively.

***The effect of TF on apoptosis of CNE2 cells***

**Figure 3:**
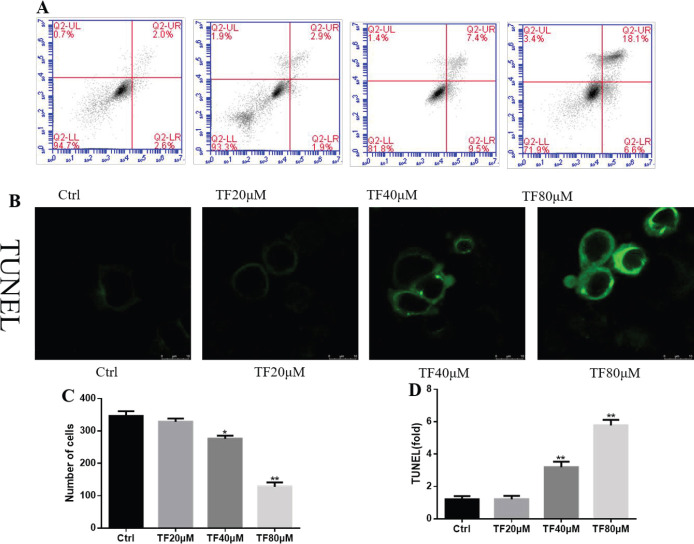
Effect of theaflavin (TF) on apoptosis of CNE2 cells detected by flow cytometry and a TUNEL assay in CNE2 cells after treatment with TF. (A) Apoptosis of CNE2 cells was analyzed by Annexin V-FTIC and PI double staining after treatment with TF at different concentrations (20, 40, or 80 μM) for 48 hr. (B) CNE2 cells were treated with TF at different concentrations (20, 40, or 80 μM) for 48 hr, fixed and treated with the TUNEL system, and images were taken from six random fields. (C-D) Data are presented as the mean ± SD from three independent experiments. *P<0.05 and **P<0.01, compared with the control group

To further explore the suppressive effect of TF, we executed an apoptosis assay by flow cytometry. Treatment with TF markedly increased the apoptosis of CNE2 cells in a dose-dependent manner. The high concentrations (40 and 80 μM) of TFs dramatically raised the apoptotic rates compared with the control group (Figures 3A and C). To detect the effect of TFs on DNA fragmentation, we performed terminal deoxynucleotidyl transferase dUTP nick end labeling (TUNEL). The results showed that the high concentrations (40 and 80 μM) of TF significantly increased the number of TUNEL-positive cells (Figures 3B and D). These results indicated that TF increased apoptotic DNA fragmentation.

***TF induced caspase-dependent apoptosis***

**Figure 4:**
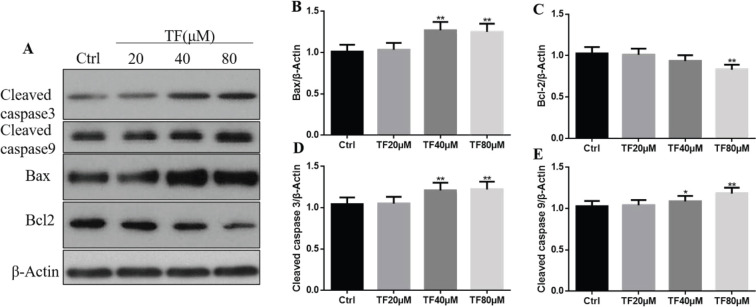
(A) Expression of cleaved caspase 3, caspase 9, Bax, and Bcl-2 proteins in CNE2 cells treated with different concentrations of TF (20, 40, or 80 μM) for 48 hr. (B) Quantification results of Western blot band intensities by ImageJ2X. Data are presented as the mean ± SD from three independent experiments. *P<0.05 and **P<0.01, compared with the control group

Apoptosis has two kinds of pathway: caspase-dependent and caspase-independent (22). Western blotting results revealed that TF regulated apoptosis in the caspase-dependent pathway. As shown in Figure 4, CNE2 cells were treated with different concentrations of TF, and apoptosis-related proteins, such as caspase 3, caspase 9, Bax, and Bcl-2, were detected. High concentrations of TF (40 and 80 μM) significantly increased the expression of caspase 3, caspase 9, and Bax and remarkably reduced the expression of Bcl-2 compared with the control group (*P<0*.*05, **P<0.01). After the treatment of TF 40 *μM, the protein *levels of caspase 3, caspase 9, and Bax were significantly elevated by115.92%,105.57%, and125.24% as compared with the control group, and TF 80 μM increased the three kinds of protein levels by 116.98% and 114.61% compared with the control group. These results indicated that TF treatment could lead to a cascade of reactions in CNE2 cells that caused apoptosis and regulated apoptosis in a caspase-dependent pathway.

***TF regulates the autophagy markers in CNE2 cells unexpectedly***

**Figure 5:**
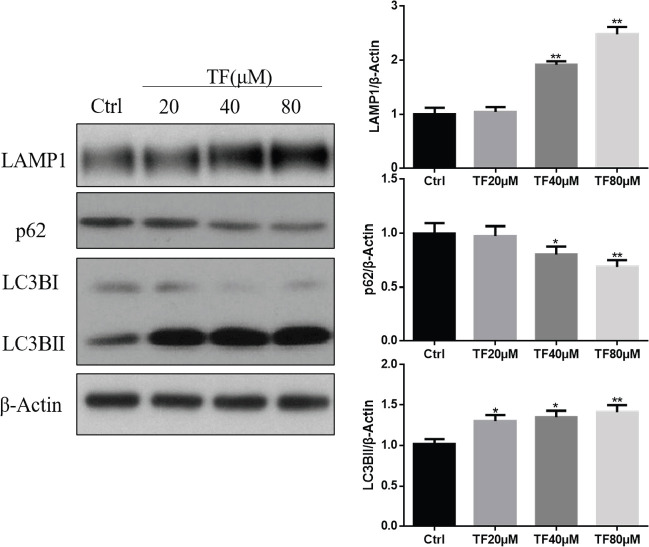
**T**heaflavin (TF) induces autophagy in CNE2 cells. (A) Western blotting results of LAMP1, p62, and LC3B. CNE2 cells were treated with TF at different concentrations (20, 40, or 80 μM) for 48 hr, and the control group was treated with a vehicle. (B) Quantification results of Western blot band intensities by ImageJ2X. The data are presented as the mean ± SD from three independent experiments. *P<0.05 and **P<0.01, compared with the control group

Generally, apoptosis-related caspase activation shuts off the autophagic process (14). Surprisingly, TF enhanced autophagy rather inhibited autophagy. As shown in Figure 5, the Western blot results showed that treatment with TF for 48 hr up-regulated LAMP1 and LC3B-II expression and down-regulated p62 expression in a dose-dependent manner. LC3B-II is the autophagosome- or phagophore-associated form of MAP1LC3B/LC3B, which is an important marker for autophagy. The level of LC3B-II significantly increased when treated with TF at a concentration of 20, 40, or 80 *μM* compared with the vehicle control (*P<0.05 and **P<0.01). The level of LAMP1 significantly increased while the level of p62 significantly decreased when treated with TF at a concentration of 40 or 80 μM compared with the control group (*P<0.05 and **P<0.01). These results indicated that TF induced autophagy.

***TF promotes autophagosome flux***

**Figure 6:**
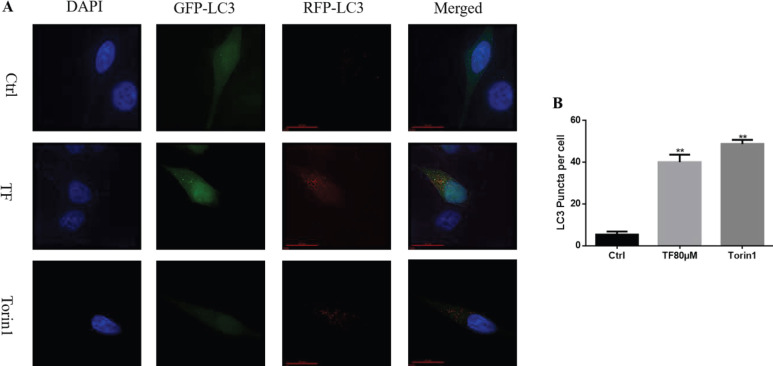
Autophagic flux represented by RFP-GFP-LC3-positive puncta was induced by theaflavin (TF) and Torin1 (as a positive control). (A) RFP-GFP-LC3 plasmid was transfected into CNE2 cells for 48 hr, and then the cells were treated with 80 μM TF and 150 nM Torin 1 for 48 hr. The control group was treated with the vehicle. (B) The number of LC3 puncta per cell was calculated and quantified. Quantification of Western blot band intensities was determined by ImageJ2X. The data are presented as the mean ± SD from three independent experiments. *P<0.05 and **P<0.01, compared with the control group

Measuring autophagy flux is essential to understanding the whole process of autophagy. To detect the autophagy flux, CNE2 cells were transfected with the RFP-GFP-LC3 plasmid. As shown in Figure 6 These cells were then treated with TF (80 μM), and Torin1 was used as a positive control. After treatment for 48 hr, the number of red puncta was found to be significantly increased in cells treated with TF compared with the control group (*P<0.05 and **P<0.01). This phenomenon indicated that TF enhanced autophagy flux.

***Inducing the autophagy effects of TF is dependent on the mTOR pathway***

**Figure 7:**
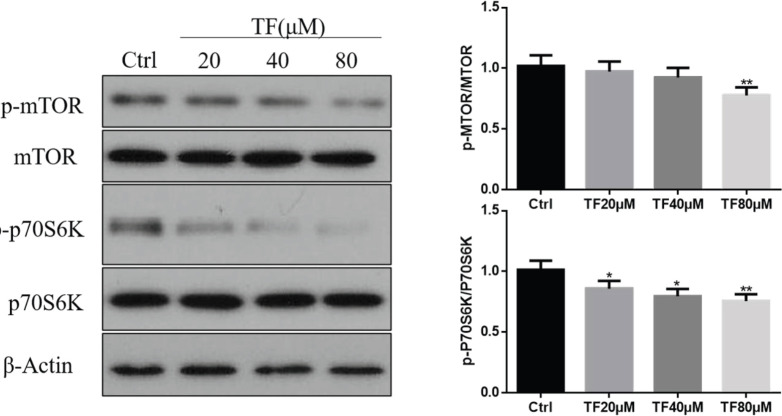
Theaflavin (TF) induced autophagy by inhibiting the mTOR pathway. (A) CNE2 cells were treated with TF at different concentrations (20, 40, or 80 μM) for 48 hr. The expression of phosphorylated (p-p70S6K) and total mTOR and p70S6K was examined by Western blotting. (B) Quantification results of Western blot band intensities by ImageJ2X. The data are presented as the mean ± SD from three independent experiments. *P<0.05 and **P<0.01, compared with the control group

The mTOR pathway is the main regulatory pathway for autophagy. To test whether TF induced autophagy through the mTOR pathway, we examined the phosphorylation of mTOR and RPS6KB1/p70S6K, which is a downstream target of the mTOR pathway. The results showed that TF remarkably reduced the phosphorylation of mTOR and RPS6KB1/p70S6K compared with the control group (Figure 7). These results suggested that TF regulated autophagy in an mTOR-dependent manner.

***The autophagy inhibitor CQ blocks the effect of TF on apoptosis-related proteins***

**Figure 8:**
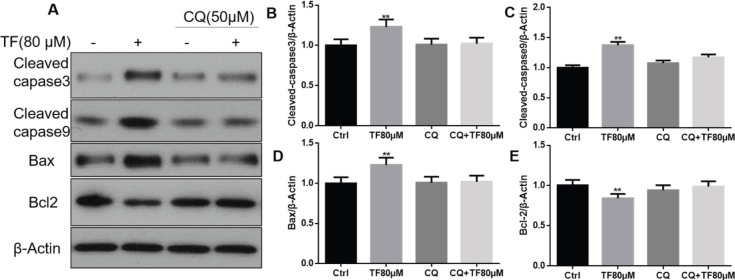
The autophagy inhibitor CQ blocks the effects of theaflavin (TF) on apoptosis-related proteins. (A) Expression of cleaved caspase 3, caspase 9, Bax, and Bcl-2 proteins in CNE2 cells treated with TF (80 μM) for 48 hr and cotreated with CQ (50 μM) for 24 hr. (B) Quantification results of Western blot band intensities by ImageJ2X. The data are presented as the mean ± SD from three independent experiments. *P<0.05 and **P<0.01, compared with the control group

After treatment with the autophagy inhibitor CQ, the effects of TF on apoptosis-related proteins were blocked. When CNE2 cells were treated with TF alone, the expression of caspase 3, caspase 9, and Bax was increased, and the expression of Bcl-2 was reduced significantly compared with the control group (*P<0.05 and **P<0.01) (Figure 8). When cotreated with CQ, these changes were all blocked. These results indicated that blocking autophagy could debilitate the effect of TF on the apoptosis process in CNE2 cells.

**Figure 9:**
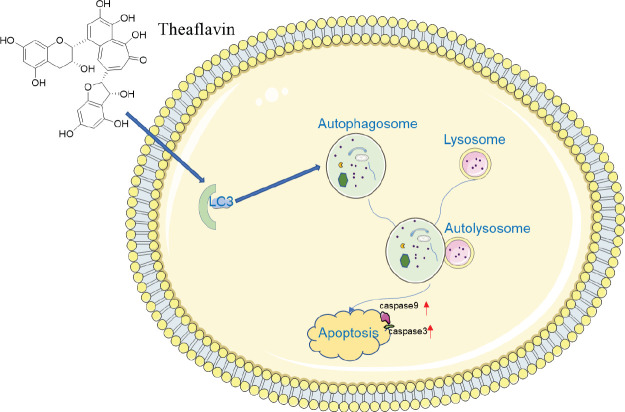
Overall illustration of the role and mechanism of theaflavin (TF) in promoting autophagy and apoptosis in CNE2 cells. TF induced autophagy rather than inhibiting autophagy through up-regulating the levels of the autophagy marker light chain 3 (LC3) and increased the levels of caspase 3 and caspase 9 to promote apoptosis

## Discussion

Nasopharyngeal carcinoma is a malignant tumor that naturally occurs in the epithelial cells of the nasopharyngeal mucosa (23). It is highly malignant and has the characteristics of early metastasis, which seriously endangers the health of people (24). The incidence is very high in some geographical and ethnic populations (25). Because of the unsatisfactory results of chemoradiotherapy and radiation therapy, it is imperative to search for less toxic and naturally-based therapies.

TF, mainly extracted from black tea, can promote programmed cell death in cancer cells(10). It may be developed as an effective drug for nasopharyngeal carcinoma. Hibasami *et al*. reported that the effective concentration of TF was 180 μM (26). Our results showed that 160 μM TF significantly suppressed CNE2 cell proliferation at different time points and that 20, 40, and 80 μM TF could generate a remarkable effect after 48 hr of treatment. Considering the mechanistic research, we used 20, 40, and 80 μM concentrations for subsequent experiments. The cell colony formation experiment confirmed the ability of TF to inhibit the proliferation of CNE2 cells.

Apoptosis is a type of programmed cell death. A variety of pathological and physiological stimuli to the cell can generate apoptosis (27). Therefore, it is useful to analyze the key signaling pathways involved in apoptosis for drug treatment (28). Bcl-2 family proteins are the key regulators of apoptosis; in this family, down-regulation of Bcl-2 expression can promote apoptosis and apoptotic function. Bax is also an important member of the Bcl-2 family. It is a heterodimer with Bcl-2, and apoptosis can be enhanced by up-regulation of Bax (29). A variety of articles have reported that Bcl-2 and Bax proteins play an important role in regulating apoptosis (30-32). Our flow cytometry results and TUNEL assay results showed that TF-induced apoptosis, reduced Bcl-2 protein expression, and significantly increased Bax protein expression, which was consistent with the results reported in previously published articles (33, 34). Caspase family proteins are essential mediators of apoptosis, which is a form of programmed cell death. Among the family members, caspase 3 is the effector of apoptosis, and it mainly catalyzes the specific cleavage of numerous important cellular proteins (35), which effectively leads to cell death. Caspase 9 is the initiator of apoptosis. It belongs to the caspase family of cysteine proteases related to apoptosis and cytokine processing, and its main function is to activate the proapoptotic protein bid (36). Our data showed that TF remarkably increased the expression of cleaved caspase 3 and caspase 9. These results indicate that treatment with TF leads to a series of caspase enzyme reactions that cause apoptosis. 

Autophagy is currently one of the hotspots in the field of tumor research, and it is also a new direction for treatment. Changes in autophagy activity can cause a variety of changes in tumor cells. Autophagy is a double-edged sword in the process of tumorigenesis and development (19). Normally, autophagy blocks apoptosis, and apoptosis shuts off the autophagy process accordingly, however, autophagy helps to induce apoptosis in some cases (14). Bai *et al*. demonstrated that zinc oxide nanoparticles induce apoptosis and autophagy in human ovarian cancer cells (37). Montani *et al*. indicated that histone deacetylase inhibitors VPA and TSA induce apoptosis and autophagy in pancreatic cancer cells (38). Zhang *et al*. showed that flavonoids inhibit cell proliferation and induce apoptosis and autophagy through down-regulation of PI3Kγ mediated PI3K/AKT/mTOR/p70S6K/ULK signaling pathway in human breast cancer cells, they found that flavonoids induced cell cycle arrest at G2/M phase, apoptosis, and autophagy (39). A study reported that a natural compound induced apoptosis and autophagy in nasopharyngeal carcinoma (40). LC3B-II is the key marker of autophagy, which is converted from LC3B-II with lipid extensions. p62 is a cargo protein and a receptor for autophagosomes. LAMP1 is a protein of the lysosomal membrane that regulates the integrity of lysosomes (41). RFP-GFP-LC3 is an uncomplicated and quantitative way to assess autophagic flux (42). Herein, TF significantly increased the expression of LAMP1 and LC3B-II and reduced p62 expression. In addition, TF remarkably promoted autophagic flux through RFP-GFP-LC3 evaluation. These results indicate that TF induces autophagy.

mTOR plays an important role in regulating autophagy. In mammalian cells, mTORC1 can phosphorylate ULK1 and prohibit the interaction of ULK1 with AMPK, which is the first step of the mammalian autophagy process (43). p70S6K is one of the substrates of mTOR and is essential for autophagy. The activity of p70S6K is maintained upstream of mTOR, and p70S6K activates autophagy, which is dephosphorylated by mTOR (44). Our results showed that TF could significantly reduce the levels of p-mTOR and p-p70S6k, thus it indicated that TF regulated the mTOR pathway. As mentioned above, TF could induce autophagy, these results demonstrated that TF induced autophagy via the mTOR pathway.

Indeed, when we used the autophagy inhibitor CQ to block the autophagy induced by TF, the expression of the apoptosis-related proteins caspase 3, caspase 9, Bax and Bcl-2, was not affected by TF. It revealed when autophagy was blocked, the cascade of apoptosis that was promoted by TF was abolished. These results indicated that TF exerted its apoptosis-inducing effects by inducing autophagy (Figure 9). These data reveal that TF performed an imperative role in promoting apoptosis* in vitro*, but the mechanism of nasopharyngeal carcinoma *in vivo* is more complex, whether TF plays an equally crucial role* in vivo* remains elusive, it needs further study in the future.

## Conclusion

Overall, TF suppressed the proliferation of CNE2 cells and induced apoptosis in a dose-dependent manner. The mechanism was through the regulation of apoptosis-related proteins and in a caspase-dependent pathway. Interestingly, TF also enhanced autophagy and lysosome biogenesis. In addition, inducing autophagy was dependent on the mTOR pathway. When autophagy was blocked, the effect of TF on apoptosis proteins was abolished. The in-depth mechanisms still need to be further explored. Our results uncover the importance of TF promoting apoptosis in enhancing autophagy rather than suppressing autophagy. This research provides a novel insight into promoting apoptosis effects of TF on nasopharyngeal carcinoma. It gives some useful implications for the treatment of nasopharyngeal carcinoma in clinical research.

## Authors’ Contributions

JX Analyzed the data, did the Western blot experiments, and wrote the manuscript. SJW Performed the immunoblotting work. SSB Did the CCK test experiment. XQW Achieved the TUNEL experiment. HG Designed and supervised the project.

## Conflicts of Interest

The authors declare no conflicts of interest.
